# Berberine increases adipose triglyceride lipase in 3T3-L1 adipocytes through the AMPK pathway

**DOI:** 10.1186/s12944-016-0383-4

**Published:** 2016-12-09

**Authors:** Dongqing Jiang, Dianhui Wang, Xianghua Zhuang, Zhanqing Wang, Yihong Ni, Shihong Chen, Fudun Sun

**Affiliations:** 1 Department of Endocrinology and Metabolism, The Second Hospital of Shandong University, Jinan, 250033 Shandong China; 2 Department of Emergency, Yantai Affiliated Hospital of Binzhou Medical University, Yantai, 264100 Shandong China

**Keywords:** ATGL, 3T3-L1 cell, HSL, Lipolysis, Obesity

## Abstract

**Background:**

Obesity is closely related to the metabolism of triacylglycerol (TG) in adipocytes. Adipose triglyceride lipase (ATGL) and hormone-sensitive lipase (HSL) are rate-limiting enzymes that control the hydrolysis of TG. Effects on ATGL and HSL to increase lipolysis may counteract obesity. Berberine (BBR) is a compound derived from the Chinese medicine plant Coptis chinensis. In the present study we show the effects of BBR on ATGL and HSL and explore the potential underlying mechanisms of these effects.

**Methods:**

The TG content in cells was measured using a colorimetric assay. The expressions of HSL, ATGL and GPAT3 were evaluated by Western-blotting. The expression of ATGL was also evaluated by real-time PCR and radioimmunoassay. Compound C, an inhibitor of AMP-activated protein kinase (AMPK), was used to explore the possible pathway that involved in the effect of BBR on ATGL.

**Results:**

TG content of differentiated 3T3-L1 cells was significantly decreased by more than 10% after treated with BBR. In differentiated 3T3-L1 adipocytes, BBR increased the expression of p-HSL and ATGL, and these effects were time-depended (*p <0.01*). The effect of BBR on ATGL expression could be abolished by Compound C which suggested that AMPK pathway was involved in the effects of BBR on p-HSL and ATGL.

**Conclusions:**

BBR could increase the expression of ATGL and therefore stimulate basal lipolysis in mature adipocytes through the associated mechanisms related to the AMPK pathway.

## Background

Obesity has become a worldwide public health problem. It is an established risk factor for metabolic diseases including type 2 diabetes, hypertension, coronary heart disease, and nonalcoholic fatty liver disease [1, 2].

In adults, obesity mainly results from an increase in the size of adipocytes, that is, the accumulation of triacylglycerol (TG) in adipocytes. There are several key enzymes that participate in the metabolic processing of TG, including glycerol-3-phosphate acyltransferase 3 (GPAT3), hormone-sensitive lipase (HSL) and adipose triglyceride lipase (ATGL). GPAT3 is the major GPAT isoform expressed in adipocytes and plays a crucial role in adipogenesis. HSL and ATGL are rate-limiting enzymes that regulate the lipolysis of TG in adipocytes [3]. They catalyze the hydrolysis of TG and effect different catabolism courses of TG [4]. ATGL primarily catalyzes the hydrolysis of TG to generate diacylglycerol (DG) and mediates the hydrolysis of triglycerides during basal lipolysis [3]. HSL mainly exhibits DG lipase and is the major lipase for catecholamine- and natriuretic peptide-stimulated lipolysis. Because of their important roles in regulating the metabolism of TG, HSL and ATGL have become common subjects of research regarding obesity and lipid metabolism.

Berberine (BBR) is a major constituent of the Chinese herb Rhizoma Coptidis. BBR is known for its antimicrobial, antiprotozoal, and antidiarrheal activity, and it is commonly used in clinical practice to treat bacterial diarrhea [5]. Recently, BBR was found to be involved in the metabolism of TG when a study showed that BBR directly decreased catecholamine-stimulated lipolysis by stimulating the expression of p-HSL in 3T3-L1 adipocytes [6]. However, it remains unclear whether BBR could directly affect ATGL in 3T3-L1 adipocytes.

AMP-activated protein kinase (AMPK) is a well-known metabolic master switch. As important rate-limiting enzymes regulating lipolysis in adipocytes, both HSL and ATGL could be modified by AMPK [6, 7]. BBR has been shown to activate AMPK, which contributes to the beneficial metabolic effects of BBR in peripheral tissues [8, 9].

Taken together, we suggest that BBR could regulate the expression of ATGL through the AMPK pathway. In this study, we tried to verify this hypothesis both in vivo and in vitro. The results of this study may help us investigate the direct effects of BBR on ATGL expression in 3T3-L1 adipocytes and the potential mechanism underlying these effects. The effect of BBR on ATGL may partially account for the anti-obesity effect of BBR.

## Methods

### Cell culture and initiation of differentiation

The 3T3-L1 cells were purchased from the American Type Culture Collection (ATCC). The 3T3-L1 cells were maintained in Dulbecco’s modified Eagle’s medium (DMEM, Gibco BRL, Gaithersburg, MD, USA) supplemented with 10% (v/v) newborn calf serum (NBS, Gibco), 100 U/mL penicillin, and 0.1 mg/mL streptomycin (KeyGEN, Nanjing, China) in a humidified 5% CO2 incubator at 37 °C. D0 was designated as the second day after the confluence of the 3T3-L1 cells. To induce differentiation, preadipocytes were treated for 2 days beginning on D0 with 0.5 mmol/L, isobutylmethylxanthine (IBMX, Sigma-Aldrich), 2.5 mmol/L dexamethasone (Dex, Sigma-Aldrich) and 8.7 mmol/L insulin (Sigma-Aldrich) in DMEM containing 10% fetal calf serum (FCS, Gibco), followed by treatment for another 2 days with insulin (10 mM) alone in DMEM containing 10% FCS. The cells were subsequently replenished with DMEM containing 10% FCS every other day. On day 12, approximately 90% of the 3T3-L1 cells had differentiated into mature adipocytes.

### Cell stimulation

Differentiated 3T3-L1 adipocytes were starved in serum-free DMEM for 24 h before stimulation. The cells were then treated with BBR (National Institutes for Food and Drug Control, Beijing, China). For experiments with Compound C (Sigma-Aldrich), the cells were pre-treated with Compound C for 1 h before stimulation with BBR according to the experimental design.

### Oil red O staining

The cells were washed twice with PBS and fixed with 4% paraformaldehyde for 15 min. After being washed twice in PBS, the cells were stained for 15 min in freshly diluted Oil Red O (Sigma) solution. The dishes were then rinsed in water and counterstained with hematoxylin for 10 s. Representative photomicrographs were captured using a system incorporated in the microscope (Axiovert 100 M Zeiss, Zeppelinstrasse, Germany).

### Triglyceride quantification

TG content was measured using a colorimetric assay (Applygen, Beijing, China). To remove glycerol, the cells were washed with PBS twice before being dissolved in standard diluent. The mixture was divided into two parts; 500 μl of the mixture was centrifuged at 2000 g for 10 min and the supernatant was collected. The absorbance of this dye is proportional to the concentration of TG present in each sample, and the absorbance was quantified at 550 nm following these reactions. The protein content of the remainder of the mixture was measured according to BCA methods. All samples were tested in duplicate, and the TG values were expressed as mmol of TG/g of protein.

### Western blot analysis

The cells were homogenized in RIPA lysis buffer containing protease inhibitors. The protein concentrations were measured using the BCA method. Proteins (100 mg) were separated on 10% SDS-PAGE gels and transferred to a PVDF membrane (Millipore, USA). The membranes were blocked in 5% (w/v) non-fat milk for 1 h and then incubated with rabbit anti-p-HSL (Cell Signaling, Beverly, MA, USA; 1:1000 dilution), rabbit anti-HSL (Cell Signaling, Beverly, MA, USA; 1:1000 dilution), rabbit anti-ATGL (Cell Signaling, Beverly, MA, USA; 1:1000 dilution), rabbit anti-p-AMPK (Thr 172), rabbit anti-AMPK (Cell Signaling, Beverly, MA, USA; 1:1000 dilution), rabbit anti-PPARγ (Millipore, USA; 1:1000 dilution) or mouse anti-β-actin (Proteintech, Chicago, IL, USA; 1:2000 dilution) primary antibodies overnight at 4 °C. The membranes were then incubated with peroxidase-conjugated anti-rabbit or anti-mouse secondary antibody for 1 h at room temperature. After washing with TBST, the immune complexes were detected using the Alpha Q Chemiluminescence System and exposed to film. The relative intensity of the target protein to HSL or to β-actin in the same sample was analyzed using the Alpha Q software.

### RNA extraction and quantitative real-time PCR analysis

Total RNA was isolated from cells using TRIzol reagent (Takara, Tokyo, Japan) according to the manufacturer’s instructions. The RT reaction was carried out using 1 mg of total RNA. Real-time PCR was performed with the Light Cycler 480 (Roche Applied Science, Indianapolis, IN). The following primer sequences were used: ATGL forward, 5′- GGATGAAAGAGCAGACGGGTAG -3′, and reverse, 5′-CGCAAGACAGTGGCACAGAG -3′, HSL forward 5′-ACTGAGATTGAGGTGCTGTC - 3′, and reverse, 5′-AGGTGAGATGGTAACTGTGAG - 3′, and β-actin forward, 5′-ACCCCAGCCATGTACGTAGC-3′, and reverse, 5′- GTGTGGGTGACCCCGTCTC-3′. β-actin was employed as an endogenous control for normalization.

### Immunofluorescence

The cells were washed with PBS, fixed with 4% paraformaldehyde for 15 min, permeabilized with 0.2% Triton X-100 for 5 min and blocked using 10% goat serum in PBS for 30 min at room temperature. They were then incubated with primary antibodies (rabbit anti- ATGL, 1:100 dilution) in blocking buffer overnight at 4 °C. Next, the cells were incubated with secondary antibodies (FITC- or TRITC-conjugated, 1:50 dilution; Zhongshan Golden Bridge Biotechnology Co. Ltd) for 1 h at room temperature. The nuclei of the cells were visualized using mounting medium with DAPI. The fluorescence levels of the cells were determined using a confocal microscope (Axiovert 100 M Zeiss, Zeppelinstrasse, Germany).

### Statistical analysis

Data are presented as the mean ± standard error of the mean (SEM). One-way analysis of variance (ANOVA) and T tests were performed using the SPSS 13.0 software package. A value of *P* <0.05 was considered to be statistically significant.

## Results

### TG content of differentiated 3T3-L1 cells was significantly decreased after BBR treatment

**Fig. 1:**
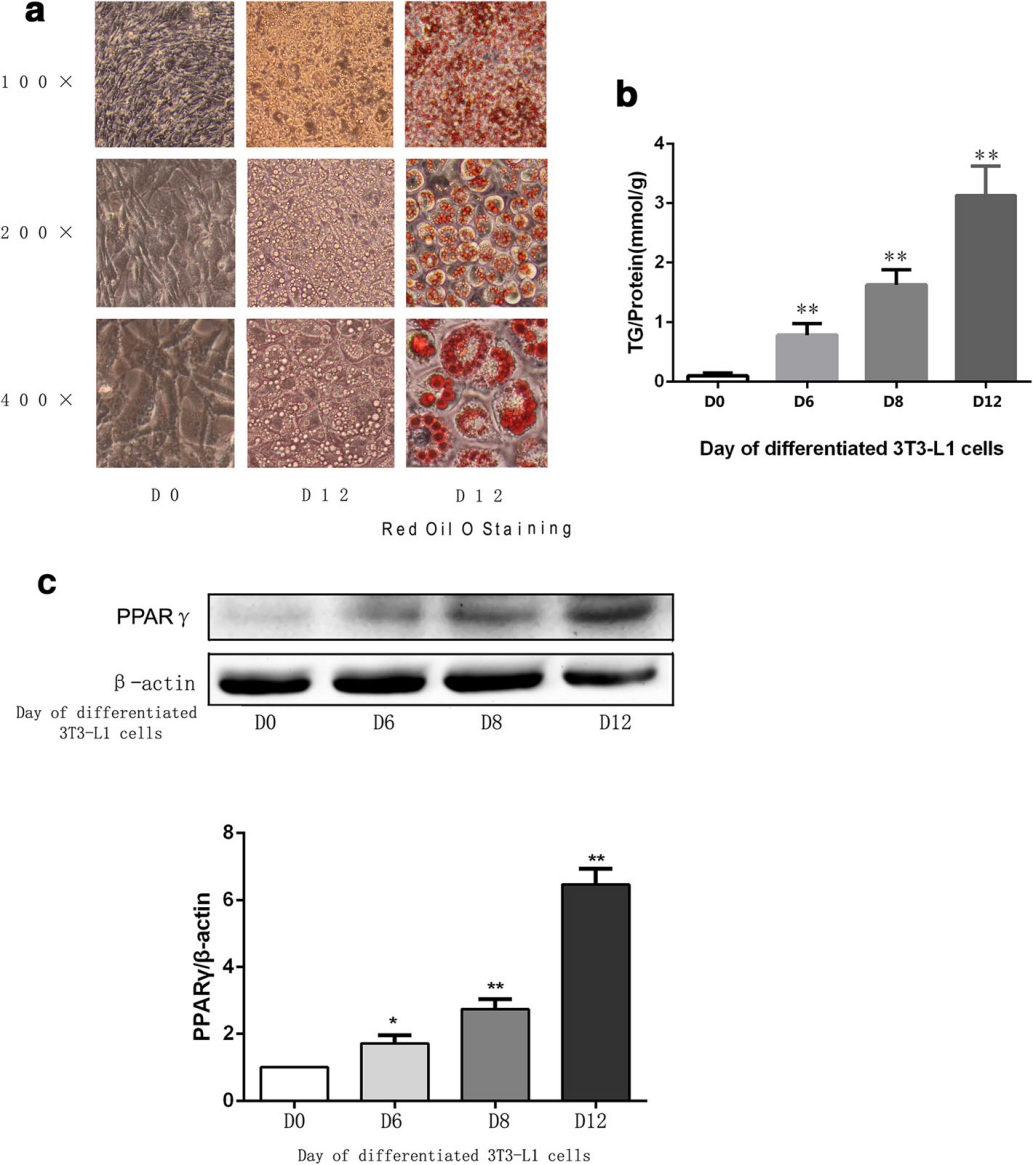
**a** Representative images of 3T3-L1 cells on D0 and D12 during the differentiated process and Oil red O staining of differentiated 3T3-L1 cells on D12. **b** TG content assay of 3T3-L1 cells on different day in the differentiated process. Intracellular TG contents were normalized by total protein. **c** 3T3-L1 cells were induced to differentiated adipocytes according to the protocol. Proteins in the cells of different days on the differentiated process were separated by SDS-PAGE and immunoblotted for PPARγand β-actin. Representative Western blot results are shown. The data were presented as the mean ± SD. * *p* <0.05, **p <0.01 versus the group of D0

**Fig. 2:**
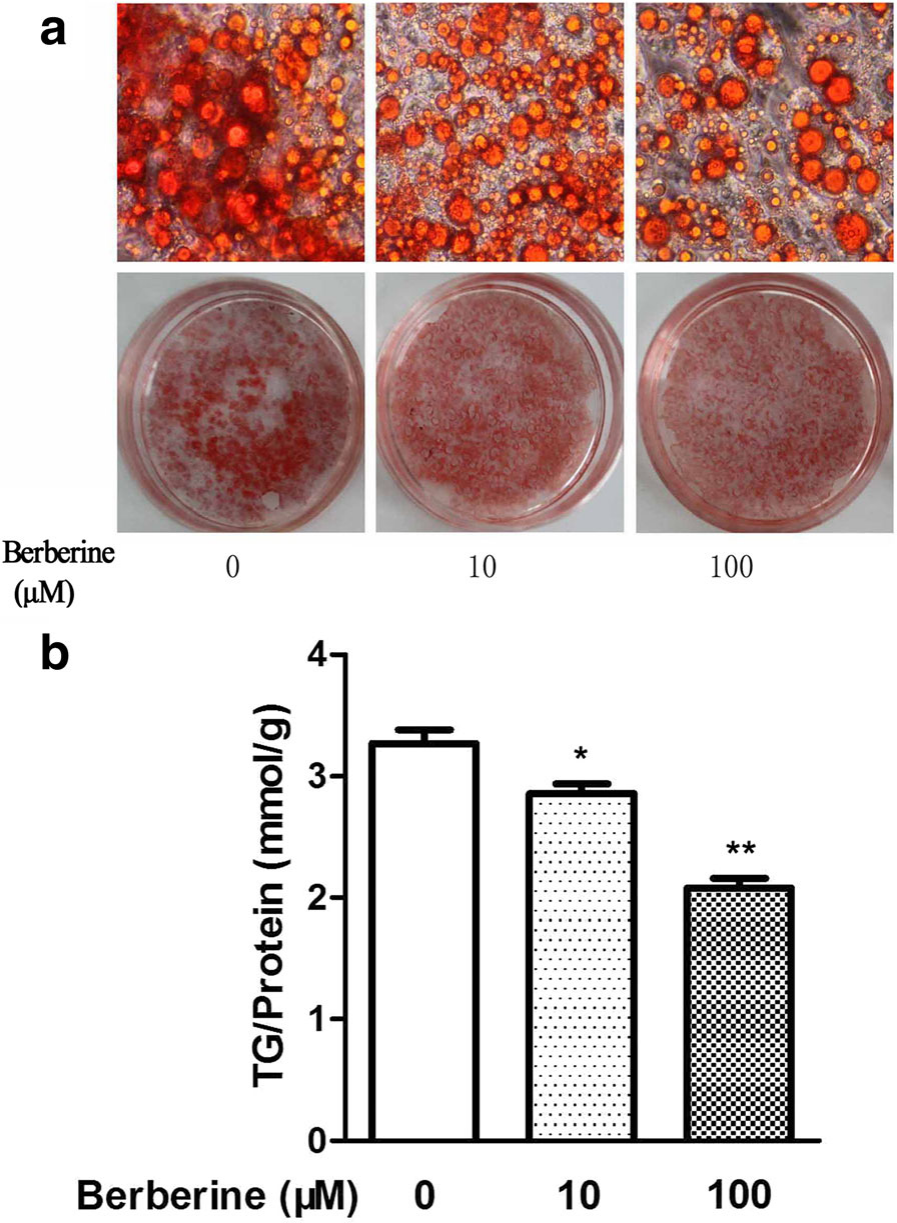
**a** Representative images of Oil red O staining and **b** TG content assay of differentiated 3T3-L1 adipocytes treated with different concentrations of BBR for 24 h. Intracellular TG contents were normalized by total protein, and both originated from one sample. The data were presented as the mean ± SD. * *p* <0.05, **p <0.01 versus the control group. Original magnification: 400 ×

The 3T3-L1 cells, derived from mouse fibroblast cells, could be induced to differentiate to adipocytes, which are widely used in research studies concerning TG metabolism and obesity. After differentiated, the TG content in differentiated 3T3-L1 cells is significantly increased, as shown in Fig. 1a. The expression of PPARγ, which is accepted as one of the standard makers of mature adipocytes, increases during the differentiation of 3T3-L1 cells, as shown in Fig. 1b. In this study, differentiated 3T3-L1 cells were used to examine the effects of BBR on TG content in vitro. Various concentrations of BBR, including 1 μM, 5 μM, 10 μM and 100 μM, have been used in studies concerning the lipolysis of differentiated 3T3-L1 cells [10]. In this study, concentrations of 10 μM and 100 μM were used. As shown in Fig. 2a, after treatment with BBR, the number of the red-stained cells had decreased, both by visual inspection and under microscopy. This effect was confirmed by the results of the TG content measured by colorimetric assay. When treated with BBR concentrations of 10 μM and 100 μM, the intracellular TG contents in differentiated 3T3-L1 cells were reduced by 12.5 and 36.5%, respectively, as shown in Fig. 2b. These results indicated that the metabolism of TG in differentiated 3T3-L1 cells may be regulated by BBR. To verify this hypothesis, the effects of BBR on the key enzymes that regulate the metabolism of TG in 3T3-L1 cells were explored.

### BBR increased the expression of p-HSL and ATGL in differentiated 3T3-L1 adipocytes

**Fig. 3:**
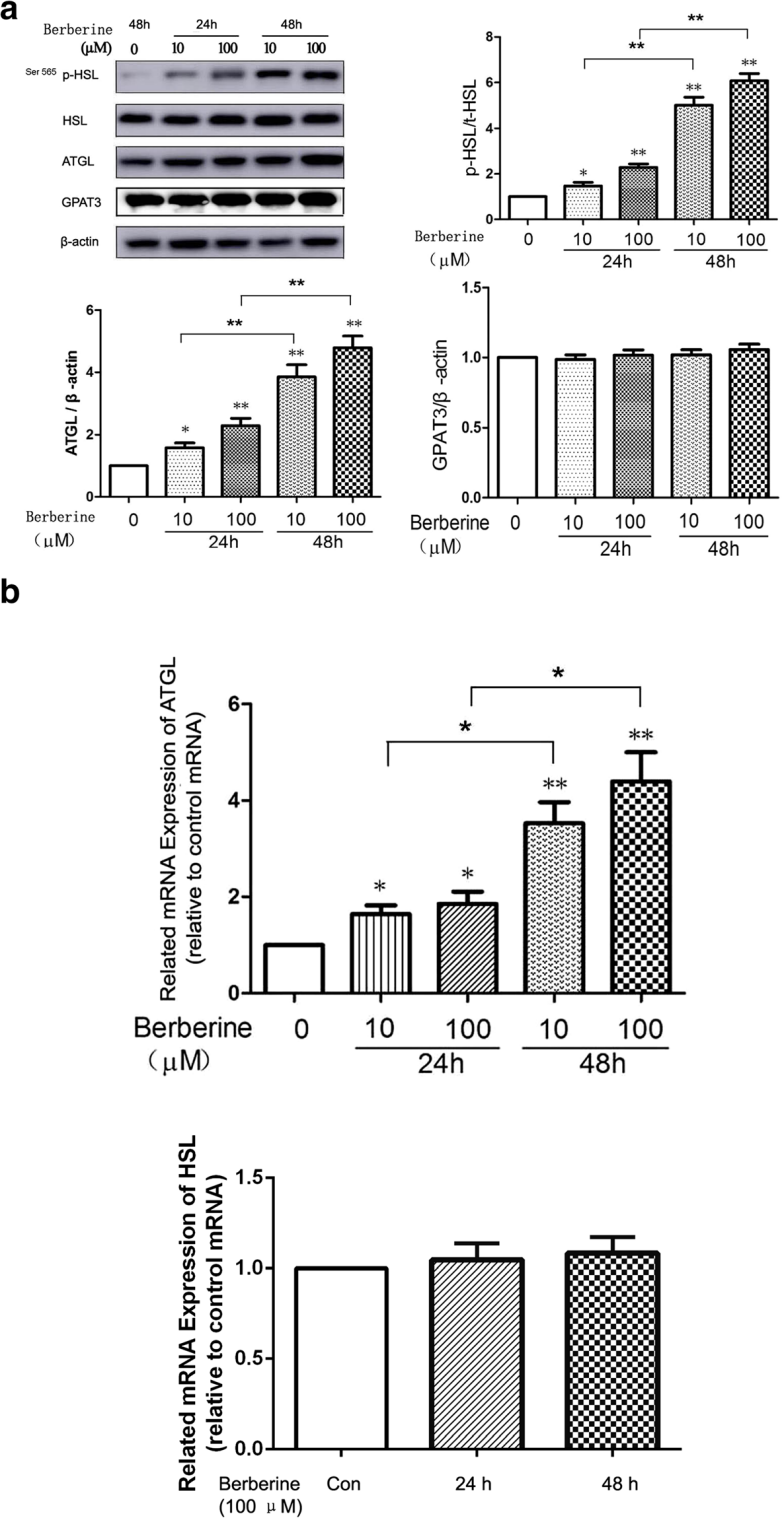
On D12, cells were treated with 10 μM and 100 μM BBR for 24 h or 48 h in serum-starved DMEM. **a** Proteins were separated by SDS-PAGE and immunoblotted for p-HSL (Ser 565), HSL, ATGL, GPAT3 and β-actin. The values of p-HSL were quantified using densitometry and normalized with HSL, while those of ATGL and GPAT3 were quantified by densitometry and normalized with β-actin. Representative Western blot results are shown. **b** Total RNA was extracted from differentiated cells treated with 10 μM and 100 μM BBR for 24 h or 48 h in serum-free DMEM. mRNA levels of ATGL and HSL were determined by real-time PCR and normalized with β-actin. Values are reported as the fold-change relative to the control group. The data are from 3 independent experiments and are presented as the mean ± SD. * *p* <0.05 versus the control group, **p <0.01 versus the control group

Currently, it is widely accepted that GPAT3, ATGL and HSL are rate-limiting enzymes involved in the metabolism of TG in adipocytes. GPAT is the key enzyme regulating the synthesis of TG [11], GPAT3 is the major GPAT isoform expressed in adipocytes, and it plays a crucial role in adipogenesis [12]. The protein expression of GPAT3 in differentiated 3T3-L1 cells was not altered significantly following treatment with BBR (Fig. 3a), and we therefore focused on HSL and ATGL in this study. In accordance with the results of Li bin Zhou [6], we found that the expression of Ser563 p-HSL, which is phosphorylated at Ser563 of HSL by protein kinase A (PKA), was not affected by BBR (data not shown), while the expression of Ser565 p-HSL, which is phosphorylated at Ser565 by AMPK, increased after treatment with BBR, as shown in Fig. 3a. It is a notable finding in this study that BBR could significantly increase the protein expression of ATGL in differentiated 3T3-L1 cells (Fig. 3a). The ATGL mRNA in differentiated 3T3-L1 cells was also increased after treatment with BBR, as shown in Fig. 3b. The effects of BBR on both Ser565 p-HSL and ATGL were time-dependent.

### AMPK pathway was involved in the effects of BBR on p-HSL and ATGL in differentiated 3T3-L1 adipocytes

**Fig. 4:**
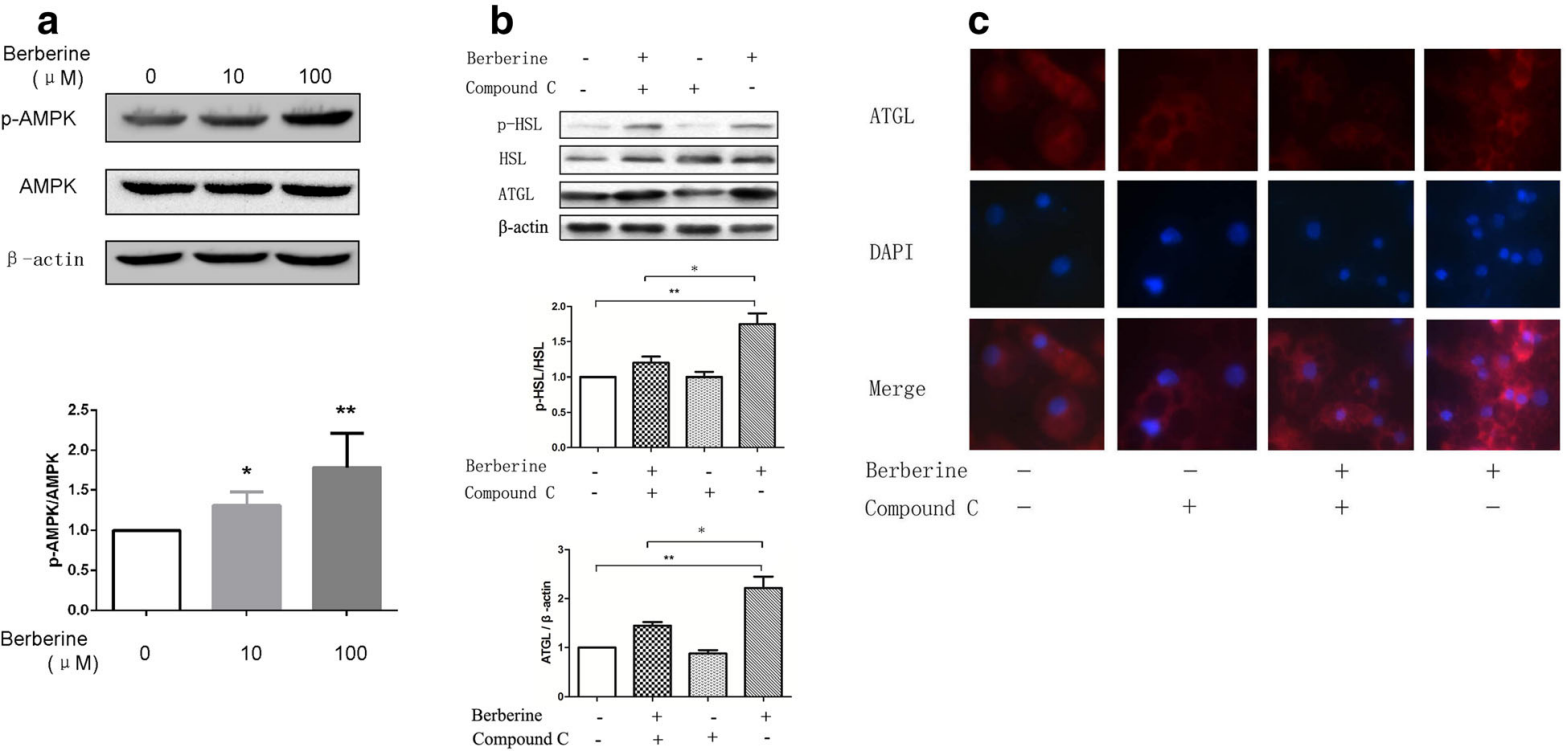
**a** On D12, cells were treated with 10 μM and 100 μM BBR for 24 h in serum-starved DMEM. Proteins were separated by SDS-PAGE and immunoblotted for p-AMPK, AMPK and β-actin. The values of p-AMPK were quantified using densitometry and normalized with AMPK. Representative Western blot results are shown. **b** On D12, the cells were stimulated with 20 μM Compound C or 10 μM BBR. After 1 h, the cells from one of the two dishes pretreated with Compound C were exposed to 10 μM BBR. All cells were treated for 24 h in serum-free DMEM. Proteins were separated by SDS-PAGE and immunoblotted for p-HSL, HSL, ATGL and β-actin. Values of p-HSL were quantified by densitometry and normalized with HSL while those of ATGL were quantified by densitometry and normalized with β-actin. Representative Western blot results are shown. Values are reported as the fold-change relative to the control group. **c** The visualization of ATGL by immunofluorescence staining (*red*). Nuclei were stained with DAPI (*blue*). Values are reported as the fold-change relative to the control group. The data are from 3 independent experiments and are presented as the mean ± SD. *p <0.05 versus the control group, **p <0.01 versus the control group. Original magnification: 400 ×

AMP-activated protein kinase (AMPK) plays a vital role in energy metabolism and is a conserved regulator of lipids metabolism. Compound C is a recognized AMPK inhibitor. In this study, the expression of p-AMPK was increased in mature 3T3-L1 adipocytes after treated with BBR, as shown in Fig. 4a. While pretreatment with compound C, which blocked the activity of AMPK, the protein expressions of ATGL and p-HSL in differentiated 3T3-L1 adipocytes treated by BBR were decreased compared with those only treated with BBR, as shown in Fig. 4b. Immunofluorescence analysis also certified these results, as shown in Fig. 4c. These results showed that the effects of BBR on ATGL and p-HSL were impaired when pretreated with compound C, which indicated that AMPK was involved in the process of BBR regulating the expression of p-HSL and ATGL.

## Discussion

The accumulation of TG in adipocytes causes obesity, and therefore decreasing the TG content in adipocytes is important for combating obesity. The present study showed that BBR directly increased the expression of ATGL in 3T3-L1 adipocytes and decreased the TG content in adipocytes. The beneficial effects of BBR on lipolysis have evoked substantial interest in the compound as a potential treatment for obesity and diseases such as diabetes.

Studies in mice have found that adipocytes decrease in size after treatment with BBR, [9] which suggests that BBR could decrease the accumulation of TG in adipocytes. Our data confirmed this effect of BBR in vitro. After treatment with BBR, lipid-drops in 3T3-L1 adipocytes reduced significantly, both in numbers and in size. To illustrate the mechanism of this effect, studies of the rate-limiting enzymes regulating TG metabolism were performed.

Activation of HSL is controlled by phosphorylation/dephosphorylation events, which are regulated by cAMP-dependent PKA and protein phosphatase, respectively [13]. PKA phosphorylates HSL at Ser563 and Ser660, which stimulates HSL to hydrolyze TG. In contrast, AMPK phosphorylates HSL at Ser565, which reduces HSL phosphorylation at Ser563 by PKA and inhibits the activity of HSL. It has been reported that neither Ser563 nor Ser660 are affected by BBR, while the expression of Ser565 increases after treatment with BBR [6], which was also shown in our study. As mentioned above, HSL is the major lipase catalyzing the rate-limiting step in stimulated lipolysis in adipocytes [3]. Taken together, all of these data suggest that do not similar to catecholamines, BBR has minimal direct impact on the acute effects of lipolysis.

ATGL is critical for the hydrolysis of TG during basal lipolysis [3]. Our study showed that the expression of ATGL in 3T3-L1 adipocytes significantly increased following treatment with BBR, which had not been reported before. This finding may explain the decrease in TG in 3T3-L1 adipocytes following treatment with BBR, and it may partially account for the weight-loss effect of BBR. That is, although the expression of p-HSL (Ser565) is also significantly increased following stimulation by BBR, the enhanced expression of ATGL increases the basal lipolysis of TG in adipocytes, causing the TG content in adipocytes to decrease, leading to weight loss.

The effects of BBR on HSL and ATGL were similar to those of thyrotropin (TSH). TSH has been found to exhibit bidirectional effects on lipolysis. Studies have found that TSH stimulated the expression of p-HSL (Ser563) to stimulate lipolysis [14], while another study found that TSH decreased the expression of ATGL to inhibit basal lipolysis [15]. We therefore speculate that the effects of BBR on the enzymes regulating lipolysis may also be bidirectional, and the detailed mechanism requires further study. In this study, the effects of BBR on p-HSL and ATGL were time-dependent rather than dose-dependent, possibly because the most effective concentration of BBR on 3T3-L1 cells was 10 μM [6], so the effect of BBR with 100 μM was not significantly different compared with that of 10 μM.

AMPK is a common target of the pathways involving energy metabolism. Both HSL and ATGL are phosphorylated by AMPK [16]. Studies have found that BBR regulated the metabolism of TG, cholesterol and glucose by regulating the expression of AMPK [17, 18]. In our study, the effects of BBR on p-HSL and ATGL were partially blocked by Compound C, which suggests that AMPK at least partially regulates the effects of BBR on p-HSL and ATGL.

Besides the effect on the metabolism of TG, BBR has also been found have the hypocholesterolemic effect [19]. The mechanism of this effect involves proprotein convertase subtilisin/kexin type 9(PCSK9) [20, 21] and LDLR [22], which is distinguished with that of statins. BBR, as an isoquinoline alkaloid, could be isolated from many medicinal herbs such as Coptidis Rhizoma, Hydrastis canadensis and Cortex Phellodendri. All of these characteristics make BBR a potential nutraceutical to treat dyslipidaemia [23].

## Conclusions

This study illustrated the novel effect of BBR in increasing the expression of ATGL in the mature adipocytes, which confirmed the statement that BBR could regulate the metabolism of TG. BBR can regulate the metabolism of both TG and cholesterol, and further studies are needed to explore the effects of BBR in regulating energy metabolism. These studies may facilitate the development of therapeutic strategies for the treatment of dyslipidemia and obesity.

## Abbreviations

AMPK: AMP-activated protein kinase
ATGL: Adipose triglyceride lipase
BBR: Berberine
DG: Diacylglycerol
FCS: Fetal calf serum
GPAT3: Glycerol-3-phosphate acyltransferase 3
HSL: Hormone-sensitive lipase
NBS: Newborn calf serum
PKA: Protein kinase A
TG: Triacylglycerol
TSH: Thyrotropin
